# Orexin-A Exerts Equivocal Role in Atherosclerosis Process Depending on the Duration of Exposure: In Vitro Study

**DOI:** 10.3390/nu12010053

**Published:** 2019-12-24

**Authors:** Narjes Nasiri Ansari, Flora Spentza, Georgios K. Dimitriadis, Aphrodite Daskalopoulou, Angeliki Karapanagioti, Gerasimos Siasos, Evi Lianidou, Athanasios G. Papavassiliou, Eva Kassi, Harpal S. Randeva

**Affiliations:** 1 Department of Biological Chemistry, Medical School, National and Kapodistrian University of Athens, 11527 Athens, Greecespentzaflora@gmail.com (F.S.); aphrodite.dask@gmail.com (A.D.); a.karapanagiwth@hotmail.com (A.K.); gsiasos@med.uoa.gr (G.S.); papavas@med.uoa.gr (A.G.P.); 2 Department of Endocrinology, King’s College Hospital NHS Foundation Trust, Denmark Hill, London SE5 9RS, UK; G.Dimitriadis@warwick.ac.uk; 3 1st Department of Cardiology, Hippokration Hospital, National and Kapodistrian University of Athens, Medical School, 11527 Athens, Greece; 4 Department of Chemistry, Analysis of Circulating Tumor Cells Lab, Laboratory of Analytical Chemistry, University of Athens, 15784 Athens, Greece; evilianidou@gmail.com; 5 Division of Translational and Experimental Medicine-Metabolic and Vascular Health, Warwick Medical School, University of Warwick, Coventry CV4 7AL, UK; 6 Human Metabolism Research Unit, WISDEM Centre, University Hospitals Coventry and Warwickshire NHS Trust, Coventry CV2 2DX, UK

**Keywords:** atherosclerosis, endothelial cells, orexin-α, MMPs, MCP-1, breast feeding, diurnal intermittent fasting

## Abstract

Orexin-A is a peptide hormone that plays a crucial role in feeding regulation and energy homeostasis. Diurnal intermittent fasting (DIF) has been found to increase orexin-A plasma levels during fasting hours, while Ramadan fasting which resembles DIF, has led to beneficial effects on endothelial function. Herein, we aimed to investigate the effects of orexin-A on the expression of molecules involved in the atherogenesis process: Monocyte chemoattractant protein-1 (MCP-1), matrix metalloproteinases 2 and 9 (MMP-2 and MMP-9) and tissue inhibitor of metalloproteinase-1 and 2 (TIMP-1 and TIMP-2), in human aortic endothelial cells (HAECs). HAECs were incubated with orexin-A at concentrations of 40 ng/mL, 200 ng/mL and 400 ng/mL for 6, 12 and 24 h. The mRNA levels of *MCP-1, MMP-2, MMP-9, TIMP-1, and TIMP-2* and orexin-1 receptor were measured by real-time qPCR. We also evaluated the MMP-2, p38, phospho-p38, NF-κΒ/p65 as well as TIMP-1 protein levels by Western blot and ELISA, respectively. MMP-2 activity was measured by gelatin zymography. Short-term 6-h incubation of HAECs with orexin-A at a high concentration (400 ng/mL) decreased *MCP-1, MMP-2* expression, *MMP-2/TIMP-1* ratio (*p* < 0.05), and MMP-2 activity, while incubation for 24 h increased MCP-1, MMP-2 expression (*p* < 0.05), MMP-2/TIMP-1 and MMP-2/TIMP-2 ratio (*p* < 0.01 and *p* < 0.05, respectively) as well as MMP-2 activity. The dual effects of orexin-A are mediated, at least in part, via regulation of p38 and NF-κΒ pathway. Orexin-A may have an equivocal role in atherosclerosis process with its effects depending on the duration of exposure.

## 1. Introduction

Atherosclerosis represents a chronic inflammatory process which involves cells of the immune system and those of the vascular wall, such as endothelial and smooth muscle cells [1]. Endothelium activation/dysfunction characterizes the initial stages of the atherogenic process, while plaque vulnerability and rupture are the result of the latter stages of atherosclerosis [2].

Endothelial cells appear to play an important role in atherogenesis via synthesis and secretion of molecules involved in all the steps of this process, such as adhesion molecules (MCP-1), metalloproteinases (MMPs) and their inhibitors (TIMPs). Thus, human aortic endothelial cells (HAECs) are a useful primary cell model to understand and study the mechanisms involved in atherosclerosis process and plaque stability [3]. MCP-1 attracts macrophages at the site of inflammation, thereby promoting the formation of the atherosclerotic plaque [4]. The activity of MMP-2 and MMP-9, which is regulated by, among others, the specific endogenous tissue inhibitors TIMP-1 and TIMP-2, is known to be associated with the initial and latter stages of the atherosclerosis process [5]. It should be mentioned that TMP-1 can be secreted by endothelial cells and circulated as soluble molecule [5].

Orexin-A, also known as hypocretin-1 (hypocretin), is a peptide hormone that plays a crucial role in feeding regulation and energy homeostasis [6]. Interestingly, orexin-A has not only been found in the central nervous system, but also in peripheral tissues, such as the gastrointestinal tract, kidney, and cardiovascular system, indicating its potential role in various physiological functions [7]. Of interest, in vitro, as well as, animal studies have shown the anti-inflammatory effects of orexin-A on cells and tissues involved in various diseases, such as multiple sclerosis, ulcerative colitis etc.; these effects are exerted via either inhibiting the synthesis and secretion of pro-inflammatory cytokines, or inducing the synthesis of anti-inflammatory cytokines and mediated via orexin receptors orexin-A receptor 1 (OX1R) and orexin-A receptor 2 (OX2R) [8,9,10].

Recently, Kim et al. demonstrated that orexin-A induced the expression of heme oxygenase-1 (HO-1) in endothelial cells (HUVECs) [11]. HO-1 is an anti-inflammatory, antioxidant, and cytoprotective enzyme that exerts beneficial effects on vascular damage and mediates the protection of endothelial cells by regulating endothelial cell activation and inhibiting endothelial cellular apoptosis [11].

Moreover, specific feeding patterns such as breast feeding in infants as well as diurnal intermittent fasting, which are characterized by a periodic increase in orexin-A plasma concentration, have been linked with beneficial effects on the atherogenesis process [12,13,14].

Herein, we aimed to investigate the effect of orexin-A on the expression of molecules involved in initial steps of inflammation, as well as in vulnerability of the atherosclerotic plaque, such as MCP-1, MMP-2, MMP-9, TIMP-1, and TIMP-2 in human endothelial cells. We also proceeded to clarify the underlying mechanisms and in particular the possible implication of NF-κΒ and p38 pathways in these effects.

## 2. Material and Methods

### 2.1. Cell Culture and Treatment

HAECs were purchased from Lonza and cultured in M200 medium (Gibco; Thermo Fisher Scientific, Inc., Waltham, MA, USA) supplemented with 10% fetal bovine serum (Gibco; Thermo Fisher Scientific, Inc.) and 10% low-serum growth supplement (Gibco; Thermo Fisher Scientific, Inc.) and 1% penicillin/streptomycin antibiotics (Invitrogen; Thermo Fisher Scientific, Inc.; Waltham, MA, USA). Cells were cultured in a cell incubator providing a humidified environment, with 5% CO_2_ and 95% air at 37 °C. Confluent fourth- to six passage HAECs were used in all experiments. Orexin-A (1455/500 U) was purchased from Tocris Bioscience and was dissolved in distilled water (vehicle). HAEC cells were serum starved for 10 h before they were treated with various concentrations of orexin-A.

### 2.2. MTS Cell Proliferation Assay

Endothelial cells were plated 16 hours (h) before treatment in a 96-well plate at a cell density of 1 × 10^4^ cells per well. Cells were then incubated with either various concentrations (40, 200 and 400 ng/mL) of orexin-A peptide or a vehicle for a period of 6, 12, 24, and 48 h, and the media was refreshed every 12 h. The percentage of viable cell was measured by using CellTiter 96 AQueous One Solution Cell Proliferation Assay (MTS) according to the manufacturer’s instructions (Promega, Southampton, UK). The colorimetric changes were measured using ELISA reader at 490 nm.

### 2.3. qRT-PCR

qRT-PCR was performed as previously described [15]. At the end of treatment, with various concentrations of orexin-A (40, 200 and 400 ng/mL) or a vehicle for a period of 6, 12 and 24 h, cells were harvested and total RNA was isolated using NucleoSpin^®^ RNA Plus (Macherey-Nagel, Düren, Germany). Quality of extracted mRNA was evaluated by nanodrop. One-thousand nanograms of RNA was reverse transcribed using LunaScript™ RT SuperMix Kit (New England Biolabs, Ipswich, MA, USA) in accordance with the manufacturer’s instructions. GAPDH was used as a normalization control. The mRNA levels of *GAPDH, MMP2, MMP-9, TIMP-1, TIMP-2, MCP-1,* and *OX1R* were measured using SYBR Green-based quantitative real-time polymerase chain reaction (qRT-PCR) protocol on a CFX96 (Biorad). The 2^−ΔΔ*C*T^ method was used to determine the expression level. Differentially expressed genes were identified through fold change filtering where a minimum of ≥2-fold change was considered significant. Oligonucleotide primers used for real time quantitative RT-PCR are as following *MCP-1* F:5^′^-AATAGGAAGATCTCAGTGCA-3^′^, R:*5*^′^-TCAAGTCTTCGGAGTTTGGG-*3*^′^, *MMP-2* F:*5*^′^-TGGCAAGTACGGCTTCTGTC-*3*^′^, R:*5*^′^-TTCTTGTCGCGGTCGTAGTC-*3*^′^, *MMP-9* F:*5^′^*-TGCGCTACCACCTCGAACTT-*3*^′^, R:*5*^′^-GATGCCATTGACGTCGTCCT-*3*^′^, *TIMP-1* F:*5*^′^-TGCGGATACTTCCACAGGTC-*3*^′^, R:*5*^′^-GCATTCCTCACAGCCAACAG*-3*^′^, *TIMP-2* F:*5*^′^-AAGAGCCTGAACCACAGGTA-*3*^′^, R:*5*^′^-GAGCCGTCACTTCTCTTGAT*-3*^′^, *OX1-R* F:*5*^′^-CAACAGGTTCTTGGTGAAG-*3*^′^, R:*5*^′^-TCAGCCTCAAACTTCCTTA*-3*^′^ *and GAPDH* F:*5*^′^-GGGTGTGAACCATGAGAAGT-*3*^′^ R:*5*^′^-CATGCCAGTGAGCTTCCCGTT*-3*^′^. All experiments were performed in triplicate.

### 2.4. SDS-PAGE and Western-Blot Analysis

Western blot analysis was performed as previously described [16]. Briefly, whole-cell lysates were prepared in lysis buffer (Cell Signaling Technology, MA, USA). Samples containing 30 μg of protein were resolved by electrophoresis gels and transferred to a nitrocellulose membrane. After blocking for 1h with 5% skim milk in PBST, membranes were incubated overnight at 4 °C with anti-MMP-2 (MAB902, R&D Systems; Minneapolis, MN, USA), NF-κB p65 (sc-8008, Santa Cruz Biotechnology, Dallas, TX, USA), p38 (D13E1-Cell Signaling Technology, MA, USA), phospho–p38 (D3F9-Cell Signaling Technology, MA, USA), and anti-β-actin (Millipore Corporation, Billerica, MA, USA) primary antibodies. Membranes were then probed with goat anti-mouse IgG-HRP (31430, Thermo Scientific) secondary antibody at room temperature (RT) for 1 h. Detection of the immunoreactive bands was performed using the Clarity Western ECL Substrate (BioRad). β-actin served as a loading control. Densitometric analysis was performed using Image J.

### 2.5. Gelatin Zymography

Gelatin Zymography activity of MMP-2 was evaluated by measuring gelatinolytic activities of pro-MMP-2 and active MMP-2. Equal numbers of HAECs (1 × 10^−6^ cells/well) were cultured in M200 medium for 24 h which was thereafter replaced with serum-free medium (starvation). After 10 h, cells were incubated with various concentration of orexin-A for 6 h and 24 h as described in Section 2.1. At the end of incubation time, the incubation medium was collected and concentrated using Amicon Ultra centrifugal filters (30 kDa-Millipore). Protein concentrations were calculated by performing Bradford assay.

Pro-MMP-2 and active MMP-2 proteins in the conditioned media were separated without prior boiling by electrophoresis through 10% sodium dodecyl sulfatepolyacrylamide gels containing 0.1% (weight/volume) gelatin (Sigma-Aldrich). The gels were incubated with 2.5% Triton X-100 for 1 h at room temperature. The gels were then incubated in the developing buffer (Invitrogen) for 16 h at 37 C. Gels were stained with 0.5% Coomassie Brilliant Blue (AppliChem, Darmstadt, Germany) and destained in a solution containing 40% methanol and 10% acetic acid. Clear zones against the blue background indicated the presence of gelatinolytic activity. Densitometrical analyses of zymographic images were performed using image J software (NIH, Bethesda, MD, USA).

### 2.6. Enzyme Linked Immunosorbent Assay (ELISA)

Cell-secreted TIMP-1 was measured with the respective ELISA kits (DTM100 X1 Timp-1 ELISA KIT, R&D Systems) contained pre-coated ELISA plates, and the assays were performed as described by the manufacturers. 

### 2.7. Statistical Analysis

Data are represented as mean ± SD. Statistical analysis was performed using Student’s *t*-test, two-tailed distribution. Statistical analysis of Real-Time PCR data was performed using the non-parametric test (Wilcoxon signed rank test) with the SPSS software v20 (SPSS Inc., Chicago, IL, USA). The minimum level of significance was set at *p* < 0.05.

## 3. Results

### 3.1. Orexin-A Did Not Affect Cell Viability/ Proliferation of HAECs after 24 Hours of Incubation

**Figure 1:**
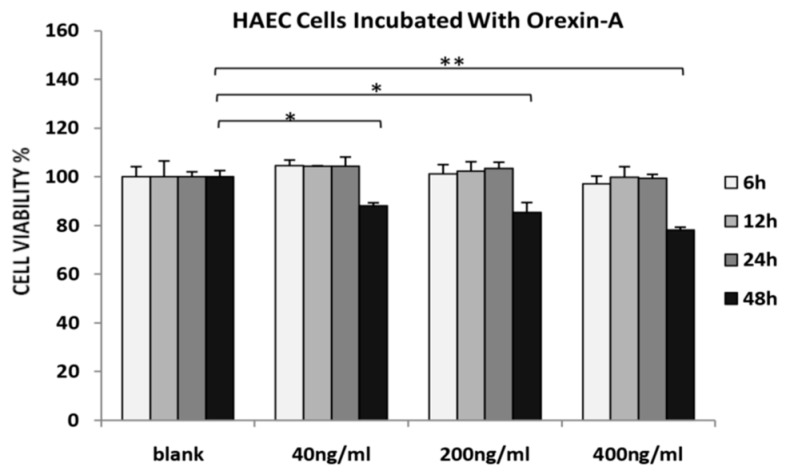
MTS assay. HAECs treated with 40, 200 and 400 ng/mL orexin-A for 6, 12, 24, and 48 h. The graphical data are represented as mean ± SD of at least three independent experiments (* *p* < 0.05, ** *p* < 0.01).

HAECs viability was decreased at all concentrations tested after incubation with orexin-A for 48 h. The maximum effect was observed at a higher concentration of orexin-A (400 ng/mL) (*p* < 0.01) as compared to either 40 or 200 ng/mL (*p* < 0.05). Incubation of HAECs with orexin-A at all concentrations tested (40, 200 and 400 ng/mL) for 6, 12 and 24 h had no significant effect on their viability (Figure 1).

### 3.2. Orexin-A Exerted A Dual Role in MCP-1, MMP-2, TIMP-1 Expression and in MMP-2 Activity

**Figure 2:**
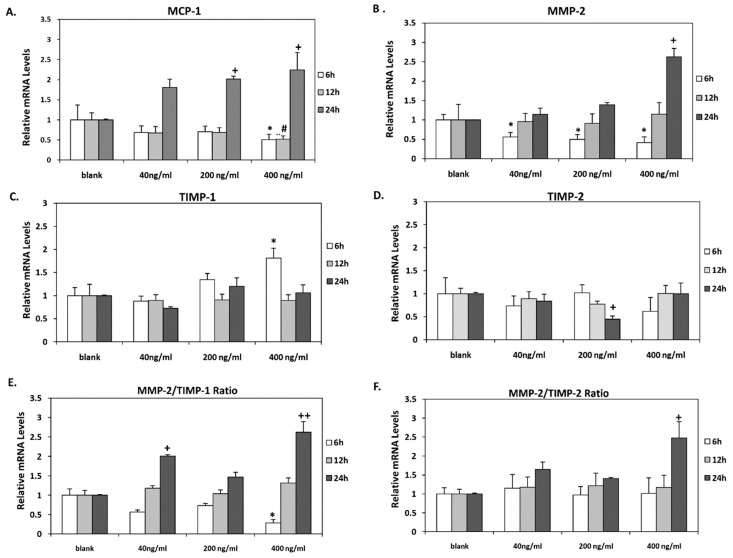
*MCP-1, MMP-2, TIMP-1,* and *TIMP-2* mRNA levels. (**A**) *MCP-1*, (**B**) *MMP-2* (**C**) *TIMP-1,* and (**D**) *TIMP-2* mRNA levels were significantly altered in HAECs after incubation with all concentrations tested (40, 200 and 400 ng/mL) for 6, 12 and 24 h. (**E**) *MMP-2/TIMP-1* mRNA ratio was significantly reduced after 6 h incubation with the highest concentration of orexin-A (400 ng/mL) while it was significantly increased after 24 h incubation with 40 and 400 ng/mL. (**F**) *MMP-2/TIMP-2* mRNA ratio was significantly increased after 24 h incubation with 400 ng/mL of orexin-A. Experiments were performed in triplicate and repeated three independent times. * significant change after 6 h incubation with orexin-A compared to control; # significant change after 12 h incubation with orexin-A compared to control; +significant change after 24 h incubation with orexin-A compared to control. Data are shown as mean ± SD (*, #, + *p* < 0.05, ++ *p* < 0.01).

Incubation of HAECs with the highest concentration of orexin-A (400 ng/mL) for 6 and 12 h resulted in significantly reduced *MCP-1* mRNA levels (*p* < 0.05) while they were significantly increased when cells were incubated with either 200 or 400 ng/mL for 24 h (*p* < 0.05) (Figure 2A).

Dose-dependent significant (*p* < 0.05) reduction of *MMP-2* mRNA was observed after short-term incubation of HAECs for 6 h at all concentrations tested. In contrast, incubation with orexin-A for 24 h stimulated MMP-*2* mRNA expression in a dose-dependent manner. However, it reached significance at 400 ng/mL (*p* < 0.05). Incubation for 12 h exerted no significant effect on *MMP-2* mRNA levels (Figure 2B).

**Figure 3:**
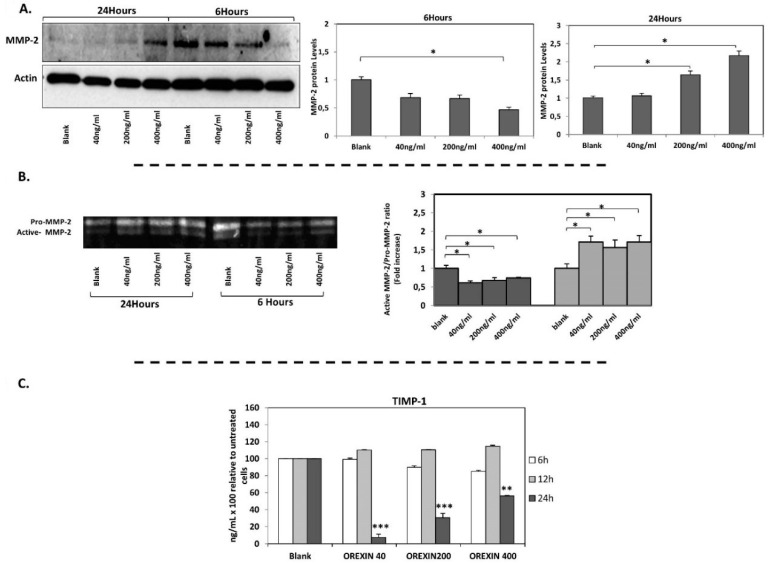
MMP-2 protein levels (Western blotting), MMP-2 gelatinolytic activity (zymogram) and TIMP-1 protein levels (Elisa). (**A**) MMP-2 protein expression was significantly decreased after incubation of cells with orexin-A for 6 h and it was induced after incubation of cells with orexin-A for 24 h. (**B**) The ratio of active MMP-2/Pro-MMP-2 was significantly reduced after 6 h incubation with various concentrations of orexin-A, while the incubation of cells with orexin-A for 24 h increased the gelatinase activity of MMP-2. (**C**) TIMP-1 protein level was significantly reduced in HAECs after incubation with all concentrations tested (40, 200 and 400 ng/mL) for 24 h. Experiments were performed in triplicate and repeated three independent times. Data are presented as the mean ± SD (*** *p* < 0.001, ** *p* < 0.01, * *p* < 0.05).

Interestingly, the effects of orexin-A on *MMP-2* mRNA expression were confirmed at protein level by Western blot analysis. Incubation of cells with orexin-A for 6 h resulted in a significant reduction in MMP-2 protein levels in a dose-dependent manner, reaching statistical significance at a higher concentration (400 ng/mL) of orexin-A (*p* < 0.05). On the contrary, MMP-2 protein was significantly induced in cells incubated with 200 ng/mL and 400 ng/mL orexin-A for 24 h (*p* < 0.05 and *p* < 0.01 respectively) (Figure 3A, Supplementary Figure S1A).

Gelatin zymography was performed to investigate the effect of orexin-A on MMP-2 activity which is essential for atherosclerotic plaque destabilization. The enzyme activity of MMP-2 was measured in HAECs incubated with all the tested concentrations of orexin-A, for 6 and 24 h. Short incubation for 6 h reduced MMP-2 gelatinase activity (*p* < 0.05) while MMP-2 activity was significantly increased after 24 h incubation with orexin-A (*p* < 0.05) (Figure 3B), albeit not dose-dependently.

*TIMP-1* mRNA expression was also increased significantly following short-term incubation (6 h) with orexin-A at the highest concentration (400 ng/mL) (*p* < 0.05) while no significant alterations in *TIMP-1* mRNA levels were observed after 12 and 24 h incubation (Figure 2C). TIMP-1 protein secreted into the culture medium of HAECs was also measured. Our results showed that incubation of cells with orexin-A for 24 h significantly decreased TIMP-1 protein expression dose dependently with the highest suppression observed at the lower concentrations of 40 and 200 ng/mL (*p* < 0.001) and lowest suppression observed at 400 ng/mL (*p* < 0.01). Of note, the incubation of cells with orexin-A at all concentrations tested for 6 and 12 h had no significant effect on TIMP-1 protein expression (Figure 3C).

Additionally, a significant reduction in *TIMP-2* mRNA expression was observed after incubation of HAECs with 200 ng/mL for 24 h, whereas its levels were not altered after 6 and 12 h incubation at all tested orexin-A concentrations (Figure 2D).

Of note, the *MMP-9* mRNA expression was not detected, at all, while *OX1R* mRNA was detected in HAECs by qPCR (Supplementary Figure S1B).

### 3.3. Orexin-A Differently Regulated MMPs/TIMPs Ratio Depending on the Duration of Exposure

Incubation of HAECs with orexin-A at a concentration of 400 ng/mL for 6 h resulted in a significant reduction (*p* < 0.05) of the *MMP2/TIMP-1* ratio. On the contrary, this ratio was significantly elevated after 24 h incubation of cells with either the lowest (40 ng/mL) or highest (400 ng/mL) concentration (*p* < 0.05 and *p* < 0.01, respectively). No significant changes in *MMP2/TIMP-1* were observed after incubation of cells with orexin-A at all the tested concentrations for 12 h (Figure 3C). The incubation of HAECs with 200 ng/mL orexin-A for 24 h resulted in a significant induction of the *MMP2/TIMP-2* ratio (*p* < 0.05) while this ratio was not significantly altered after 6 and 12 h incubation with orexin-A (Figure 2E,F).

### 3.4. Orexin-A Can Either Activate or Deactivate p38 and NF-κB p65 Pathway Depending on the Duration of Exposure

In order to investigate the underlying mechanism of orexin-A regulation of MCP-1 and MMP-2 expression in HAECs, we measured the protein levels of activated members of the p38 (phosphorylated-p38) MAPK signaling pathway, total p38 as well as the levels NF-κB p65.

Orexin-A treatment (200 and 400 ng/mL) for 6 h resulted in a significant decrease in NF-κB p65 protein levels (*p* < 0.05 and *p* < 0.01, respectively) while the 24 h incubation with both concentrations resulted in higher NF-κB p65 protein levels compared to untreated cells (*p* < 0.05).

Total p38 protein levels were significantly reduced in cells treated with either 200 or 400 ng/mL (*p* < 0.05) as early as 6 h after incubation. In contrast, cells incubated with orexin-A for 24 h exhibited increased total p38 protein expression at all tested concentrations, although it was only significant in 200 ng/mL of orexin-A (*p* < 0.05).

**Figure 4:**
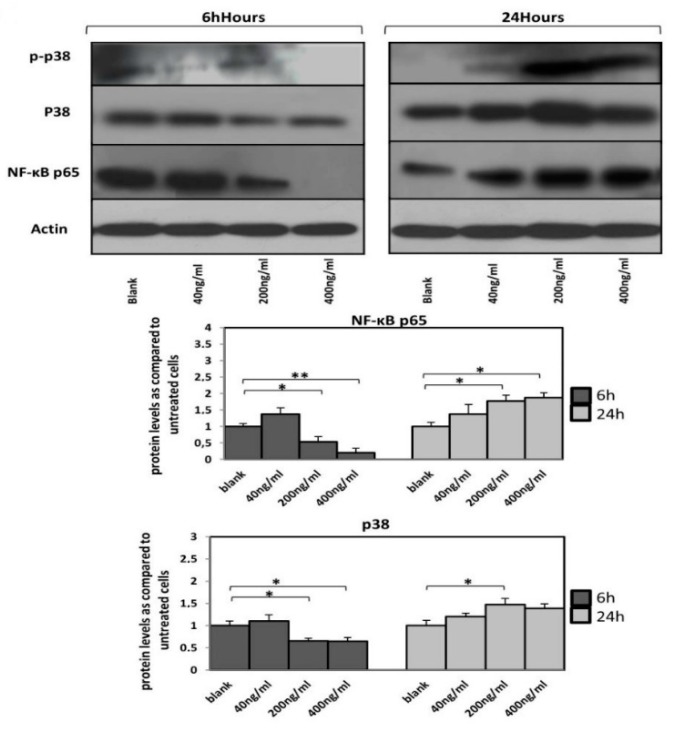
A time-dependent expression of NF-κB p65, p38 and phospho-p38 protein levels. Reduced NF-κB p65 protein levels as well as p38 MAP kinase activation in HAECs after incubation with orexin-A for 6 h. Twenty-four hour incubation with orexin-A resulted in increased NF-κB p65 protein levels as well as p38 MAP kinase activation. A representative blot out of at least three independent experiments is shown at the top; bottom, densitometric analysis. Data are mean ± SEM (fold increase); data are presented as the mean ± SD, ** *p* < 0.01, * *p* < 0.05).

Interestingly, 6h after treatment, the level of phospho-p38 was dose-dependently decreased and not detected at all in cells incubated with the highest concentration (400 ng/mL) of orexin-A, while an increase in the phospho-p38 was observed after treatment with orexin-A for 24 h (Figure 4).

## 4. Discussion

The existing literature on the role of orexin-A in the regulation of molecules implicated in the atherogenesis process is scarce. Recently, Kim et al. found that incubation of HUVECs with orexin-A at concentrations of 0.1–0.3 μM and 0.2 μM, similar to those used in our study, for 2 and 8 h, respectively positively affect the expression and activity of HO-1, which is an anti-inflammatory molecule [11].

Herein, we demonstrated that short-term incubation (6 h) of HAECs with orexin-A causes a dose-dependent decrease in *MCP-1* gene with a significant reduction at the higher concentration. Messal et al., using an animal model of ulcerative colitis, have demonstrated the anti-inflammatory effects of orexin-A via decreasing the expression of pro-inflammatory cytokines, amongst them MCP-1 in immune cells, an effect mediated by OX1R [9]. Similar effects of orexin-A on *MCP-1* expression have been reported by Fatemi et al. using an animal model of experimental autoimmune encephalomyelitis (EAE) [10]. Zhang et al. found that orexin-A at concentrations of 2.5 and 5 μΜ suppresses MAP kinase p38 phosphorylation and NF-κB activation via its receptor OX1R in HUVEC endothelial cells [17]. Interestingly, there are data indicating that MCP-1 expression is regulated by p-38 MAPK and NF-κB signaling in HUVECs [18]. Taking into account our results, we could hypothesize that orexin, acting through its receptor OX1R, could decrease the expression of *MCP-1* via inhibiting the MAP kinase p38 phosphorylation and NF-κB activation in HAECs. Notably, in line with our findings, Zhang et al. reported this effect during the short-term (6 h) incubation of HUVEC with orexin-A, thus strengthening our hypothesis [17].

Moreover, our data demonstrate that, in contrast to the favorable effects of short term incubation on *MCP-1* expression, incubation with orexin-A for a longer period led to an increase in *MCP-1* mRNA expression which was more pronounced over 24 h, suggesting a possible negative effect on the progression of atherosclerosis. We hypothesize that orexin-A differentially affects the activation of transcription factors implicated in MCP-1 regulation, such as NF-κΒ and/or p38, in a time-dependent manner. Of note, dual regulation of NF-κB activity: short (1–3 h) vs. long (12–24 h) duration of NF-κB inhibition, has been found to result in neuroprotection and aggravate damage of neuron cells, respectively [19]. According to our results, orexin-A appeared to affect total expression as well as phosphorylation of p38 equivocally; it decreased total p38 expression during short time (6 h) incubation while it increased it during a longer incubation of 24 h. Moreover, orexin-A was found to increase the phosphorylation of p38 during long incubation of 24 h and reduced it during short incubation (6 h) with the most robust effect at the highest concentration. The NF-κB pathway implication in mediating the orexin-A effects was also demonstrated. Indeed, orexin-A regulated the NF-κB pathway since it increased p65 expression during 24-h incubation and decreased its expression during 6-h incubation. Notably, the p65 expression was almost totally suppressed after 6-h incubation of HAECs with the higher concentration of orexin-A (400 ng/mL), where we observed the more pronounced effects regarding the suppression of MCP-1 expression as well as the TIMP-1/MMP-2 ratio and MMP-2 activity. All the above suggest the involvement of NF-κΒ and p38 pathways in mediating, at least in part, the dual effects of orexin-A on the expression of molecules implicated in the atherosclerosis process.

Interestingly, it has been reported that MCP-1 increases MMP-2 expression in human endothelial cells, and MMP-9 expression in human smooth muscle cells [20,21]. To this end, we studied the effect of orexin-A on the expression of MMP-2 and MMP-9. MMP-9 mRNA was not detected either under basal conditions—a result which is in line with previous reports [22]—or after incubation with orexin-A.

Concerning MMP-2, we demonstrated favorable effects of orexin-A following short-term incubation (6 h), while longer incubation periods led to an increase of MMP-2 mRNA levels, with the more pronounced effect seen at 24 h. Of note, the evaluation of MMP-2 protein expression confirmed these results. Apart from the increased expression of MMP-2, orexin-A also attenuated the MMP-2 activity in short-term incubation, while it increased the activity of MMP-2 after 24 h. To the best of our knowledge, there are no data on the regulation of MMP-2 expression and activity by orexin-A in any type of cell or tissue.

Since the ratio of MMPs/TIMPs is more indicative of MMPs activity, we evaluated the *MMP-2/TIMP-1* and *MMP-2/TIMP-2* mRNA ratio. We found that the *MMP-2/TIMP-1* ratio was decreased by orexin-A after a short period 6-h incubation at all concentrations but statistically significant at the highest (400 ng/mL). It should be noted that this effect was attributed mainly to the suppressive effect of orexin-A on *MMP-2*. On the contrary, the *MMP-2/TIMP-1* ratio was dose-dependently increased during the longer incubation period of 24 h. This unfavorable effect was attributed to both the induction of *MMP-2* and a decrease of *TIMP-1* mRNA levels resulting in respective protein concentration changes; actually, TIMP-1 protein reduction by orexin-A was higher compared to mRNA reduction, implying post-transcriptional modifications.

In the same direction, longer incubation of 24 h, also increased the MMP*-2/TIMP-2* ratio, which along with the increased MMP-2 activity, strengthened the possible detrimental effects of orexin-A in plaque stability.

According to our data, the effects of orexin-A could be mediated via OX1R, which is known to preferentially bind orexin-A, since *OX1R* mRNA was detected in HAECs. Zhang et al. have documented that the OX1R expressed in HUVECs and silencing of *OX1R* completely abrogated the inhibitory role of orexin-A in THP-1 cells attachment [17].

It is well established that poor nutrition in childhood produces long-life effects predisposing to chronic diseases during adulthood, such as atherosclerosis [23,24]. It has been demonstrated that breast-feeding of infants exerts lipid-lowering and anti-hypertensive effects in later life [25,26,27,28]. Moreover, breast-fed infants demonstrate a significant postprandial periodic increase in orexin-A plasma concentration compared to fasting. This fluctuation of orexin-α was not observed during total parenteral nutrition or in infants fed highly hydrolyzed diet. Actually, total parenteral nutrition led to a continuous stimulation and lack of fasting/postprandial modulation of orexin-A, which could be implicated in impaired development of children [12]. Taking into account our findings, it could be speculated that periodic and repetitive orexin-A secretion during the breast feeding period could be associated with a decreased risk of atherosclerosis in adulthood.

Moreover, diurnal intermittent fasting (DIF) was found to increase orexin-A plasma levels during fasting hours [13] while Ramadan fasting, which resembles DIF, led to beneficial effects on endothelial function through modulating serum asymmetric dimethylarginine (ADMA) and nitric oxide (NO) levels [14]; the above findings strengthen the hypothesis that the pattern of orexin-A secretion could be involved in the atherogenesis process.

In summary, the present study demonstrated that orexin-A could inhibit the initiation of the atherosclerosis process via reduction of the expression of *MCP-1* by endothelial cells. Moreover, orexin-A could prevent atherosclerotic plaque destabilization and rupture, by decreasing the *MMP-2/TIMP-1* ratio and MMP-2 activity. Interestingly, these favorable effects were demonstrated only during a short incubation period of 6 h, while there was no impact or detrimental effect on the expression of these molecules during the longer incubation period of 24 h and were mediated, at least partially, through equivocal regulation of p38 and NF-κB pathway. This emphasizes the importance of the duration and the pattern of the orexin-A effect on cells involved in the atherosclerotic process which could be extrapolated to the feeding behavior in humans.

## 5. Conclusions

Orexin-A may have an equivocal role in atherosclerosis process/plaque stability with its effects depending mainly on the duration of exposure. Further studies are warranted to confirm these effects in atherosclerosis animal model and humans, and to shed more light on the mechanism of action of orexin-A in human aortic endothelial cells and in other cells involved in the atherogenesis process, providing new possibilities for therapeutic approaches.

## Supplementary Materials

The following are available online at https://www.mdpi.com/2072-6643/12/1/53/s1, Figure S1: MMP-2 protein levels and OX1R mRNA expression in HAECs. (**A**). MMP-2 protein levels (measured by western blotting) is reduced by extension of starvation time in HAECs. (**B**). OX1R mRNA is detected in HAECs by qPCR.

## Abbreviations

HAECs	human aortic endothelial cells
HUVECs	human umbilical vein endothelial cells
OX1R	Orexin receptor type 1
GAPDH	glyceraldehyde-3-phosphate dehydrogenase
MMP-2	matrix metalloproteinase-2
MMP-9	matrix metalloproteinase-9
MCP-1	monocyte chemoattractant protein 1
TIMP-1	tissue inhibitor of metalloproteinases-1
TIMP-2	tissue inhibitor of metalloproteinases-2
CCL-2	chemokine (C-C motif) ligand 2
HO_1	heme oxygenase-1
