# Supplementary material for: Orexin-A Exerts Equivocal Role in Atherosclerosis Process Depending on the Duration of Exposure: In Vitro Study
Source: Nutrients. 2019 Dec 24;12(1):53. doi: 10.3390/nu12010053 (PMC7019720; doi:10.3390/nu12010053)
Supplement: Supplementary file 1 [file nutrients-12-00053-s001.pdf]

Supplementary

S.1.A. S.1.B.

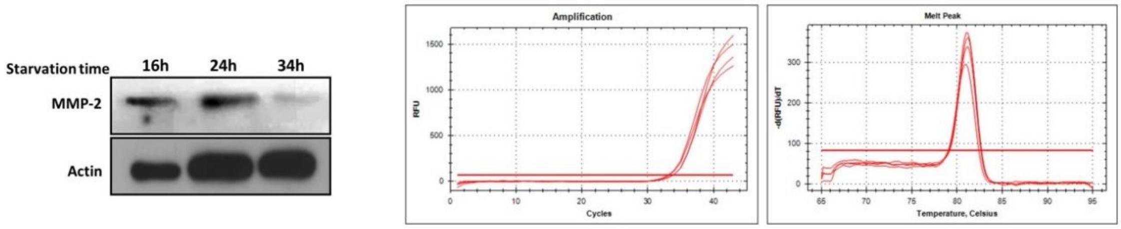

Figure S1. MMP-2 protein levels and OX1R mRNA expression in HAECs. (**A**). MMP-2 protein levels (measured by western blotting) is reduced by extension of starvation time in HAECs. (**B**). OX1R mRNA is detected in HAECs by qPCR.
